# Pentoxifylline Protects the Rat Liver Against Fibrosis and Apoptosis Induced by Acute Administration of 3,4-Methylenedioxymethamphetamine (MDMA or Ecstasy)

**Published:** 2013-08

**Authors:** Shabnam Movassaghi, Zahra Nadia Sharifi, Farzaneh Mohammadzadeh, Mansooreh Soleimani

**Affiliations:** 1 Department of Anatomy, Islamic Azad University Tehran Medical Branch, Tehran, Iran; 2 Department of Anatomy, Tehran University of Medical Sciences, Tehran, Iran; 3 Cellular and Molecular Research Center, Iran University of Medical Sciences, Tehran, Iran; 4 Department of Anatomy, Iran University of Medical Sciences, Tehran, Iran

**Keywords:** Apoptosis, Fibrosis, Liver, MDMA, Pentoxifylline

## Abstract

***Objective(s):*** 3,4-Methylenedioxymethamphetamine (MDMA) is one of the most popular drugs of abuse in the world with hallucinogenic properties that has been shown to induce apoptosis in liver cells. The present study aimed to investigate the effects of pentoxifylline (PTX) on liver damage induced by acute administration of MDMA in Wistar rat.

***Materials and Methods:*** Animals were administered with saline or MDMA (7.5 mg/kg, IP) 3 times with 2 hr intervals. PTX (200 mg kg, IP), was administered simultaneously with last injection of MDMA in experimental group.

***Results:*** The concomitant administration of pentoxifylline and MDMA decreased liver injury including apoptosis, fibrosis and hepatocytes damages.

***Conclusion:*** Our results showed for the first time that PTX treatment diminishes the extent of apoptosis and fibrosis caused by MDMA in rat liver.

## Introduction

3,4-Methylenedioxymethamphetamine (MDMA or ecstasy), is a psychoactive recreational hallucinogenic substance and a major worldwide drug of abuse )^1^).

The liver may be considered as the most important organ in drug toxicity for two reasons: 1- it is functionally interposed between the site of absorption and the systemic circulation and is a major site of metabolism and elimination of foreign substances; 2- these features also render liver a preferred target for drug toxicity. Drug-induced liver injury (DILI) therefore poses a major clinical problem (2).

Acute exposure to MDMA alone or in combination with other substances, can damage several organs such as the heart, liver, kidney, and brain and can be fatal. The life threatening clinical manifestations of MDMA toxicity also include acute hepatic damage (3-5), Despite the well-established toxicities associated with its abuse, MDMA is the second most common cause of liver injury in people under the age of 25 (1) and has been shown to produce cell necrosis and fibrosis in the liver (6).

 Although many reports have demonstrated MDMA-induced liver damage (7-9), the underlying mechanism is poorly understood. One proposed mechanism is that the reactive metabolites of MDMA are responsible for hepatotoxicity. Increased production of ROS and/or toxic oxidation products with GSH depletion may be responsible for liver damage (10-12).

Increased oxidative stress causes organ damage and apoptosis. Mitochondria, fat and anti-oxidant defense are known to be a major target of increased oxidative/nitrosative stress upon exposure to toxic compounds and/or in pathological conditions (13, 14). It is possible that MDMA and/or its reactive metabolites inhibit the mitochondrial function by directly interacting with mitochondrial proteins (15). In addition, MDMA and metabolites can indirectly cause mitochondrial dysfunction through increased oxidative/nitrosative stress (16, 17). 

Many liver injuries such as jaundice, hepatomegaly, centrilobular necrosis, hepatitis and fibrosis may be caused by MDMA and it is clarified that this drug can induce hepatocellular damage (9).

Pentoxifylline (PTX), a methylxanthine derivative and a nonspecific type 4 phosphodiesterase inhibitor, is clinically used in the treatment of lower extremity claudication. The mechanisms underlying its beneficial effects seem to be related to alterations in cellular functions and to the improvement of microcirculatory perfusion in both peripheral and cerebral vascular beds (18-19).

Serum level of cytokines such as tumor necrosis factor (TNF)-α, interleukin (IL)-1, IL-6 and IL-8 are elevated in acute alcoholic hepatitis (20). Recently, PTX, with combined anti-inflammatory (TNF-α inhibition) and antifibrogenic properties, has been found to be useful in patients with acute alcoholic hepatitis (21, 22). 

The objectives of the present work were to investigate the possible protective effects of pentoxifylline on a hepatotoxicity due to high dose of MDMA.

## Materials and Methods

***Animals and chemicals***

Adult male Wistar rats (12-13 weeks-old) weighing (250-300 g) from Pharmacology department of Tehran University of Medical Sciences were used in all experiments. The rats were housed under a 12 hr light/dark cycle. Animals were allowed free access to food and water.

All chemicals were purchased from Sigma except PTX powder that was gifted kindly by the Amin Pharmaceutical Co (Esfehan-Iran) and pure MDMA was gifted by Dr Foroumadi, Faculty of Pharmacy and Pharrmaceutical Sciences Research Center, Tehran University of Medical Sciences. 

***Experimental groups and drug***

Animals (n=24) were divided randomly into 4 groups as described below:

1- Control group: Animals were euthenized after 2 weeks.

2- Ecstasy group: 7.5 mg/kg MDMA was injected intraperitoneally (IP) three times every 2 hr.

3- Experimental group: 7.5 mg/kg MDMA was injected intraperitoneally (IP) three times every 2 hours. After the last injection of MDMA, 200 mg/kg PTX was injected IP.

4- Vehicle group: 7.5 mg/kg MDMA was injected intraperitoneally (IP) three times every 2 hr. After the last injection of MDMA, normal saline was injected IP.

Animals were sacrified after 2 weeks. Livers were removed for histological assessment (H&E and trichrom-Masson) and TUNEL technique for apoptosis. 

***Histopathology***

After fixation in 4% paraformaldehyde, samples were embedded in paraffin. Sections of 5 micron in thickness were subjected to haematoxylin and eosin and Masson’s trichrome staining and 3 µm thickness for TUNEL technique prior to examinations. Hepatic morphology was assessed by light microscopy.

**Figure 1:**
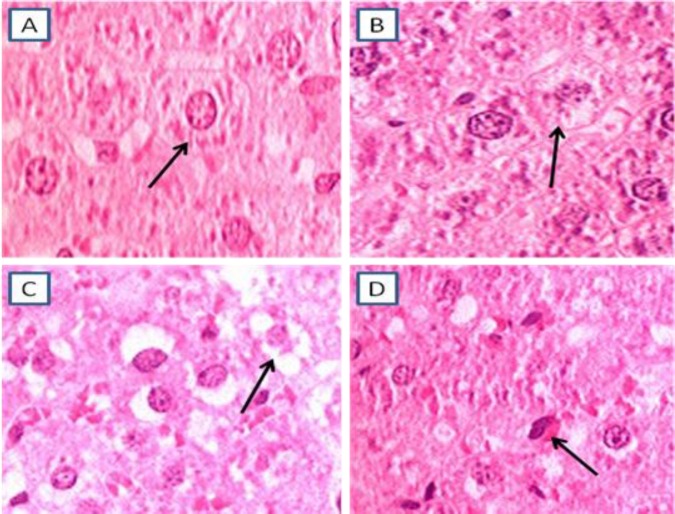
Different grades of hepatocyte damage, Arrowes' heads show hepatocytes. A) normal, B) mild C) Moderate, D) sever (H&E ×1000)

**Figure 2:**
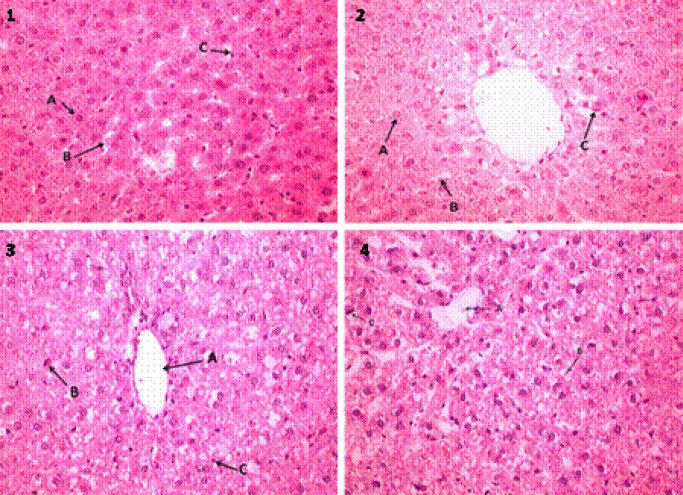
1) Control liver tissue section. A: normal Hepatocyte, B: Perisinusoidal space, C:Kupffer cell. 2) Exprimental group1. A: Hepatocyte, B: Perisinusoidal space, C: Kupffer cell, 3) Ecstasy group. A: Lobular central vein, B: Hepatocyte with severe damage, C: Lymphocyte, 4) Vehicle group. A: Lobular central vein, B: Hepatocytes cells with moderate damage, C: Hepatocyte with severe lesion. (H&E ×400)

A total of five sections for each liver tissue sample were observed under ligth microscope.

In H&E staining, damaged hepatocytes graded as 1= normal 2= mild damage (swollen and pale cytoplasm) 3= moderate damage (vacualated cytoplasm) and 4= sever damage (pyknotic nucleus and eosinophil cytoplasm) (Figure 1).

***TUNEL staining***

For TUNEL staining, paraffin blocks were cut into 3 µm thickness coronal sections. To detect apoptotic cells, TUNEL staining was performed using an In Situ Cell Death Detection Kit (Roche, Mannheim, Germany) according to the manufacturer’s protocol. 

**Table 1:** Extent of fibrosis in various groups.The values represent the mean±SD. Extents of fibrosis were graded as 0= no increase; 1= slight increase; 2=moderate increase; 3=distinct increase; 4=severe increase. the scores were combined according to the Knodell’s scoring method

Groups	Fibrosis score	Knodell score
Control	0	0
Ecstasy	2.2±9.96	6.7±1.26
Experimental (PTX)	1.1±0.82	1.5±0.58
Vehicle	2.5±0.58	7±0.82

Briefly, the sections were deparaffinized in xylol, rehydrated by successive series of alcohol, washed in phosphate-buffered saline (PBS) and deproteinized (or permeabilized) by proteinase K (20 µg/ml) for 30 min at room temperature. The sections were rinsed and incubated with 3% H_2_O_2_ in methanol for 10 min in the dark to block endogenous peroxidase (POD) and the sections incubated in the TUNEL reaction mixture for 60 min in 37°C at humidified atmosphere and rinsed with PBS. Sections were visualized using converter-POD for 30 min in 37°C at humidified atmosphere in the dark and rinsed with PBS, and 50-100 µl DAB substrate [diaminobenzidine (DAB)] was added and rinsed with PBS. 

***Masson staining***

Extents of fibrosis were graded as 0=no increase; 1=slight increase; 2=moderate increase; 3=distinct increase; 4=severe increase. Extents of periportal bridging, intralobular degeneration, portal inflammation and fibrosis were also graded according to the Knodell’s scoring method (25). 

 All slides were mounted by cover slip and analyzed by light microscope. Images were taken at x400 magnification with a light microscope (Olympus AX-70) and the number of TUNEL-positive cells and hepatocyte in different stages of damage (H&E) analyzed by image tool 2 software. All counts were performed blindly.

***Statistical analysis***

The results are given as the mean±SD. The significant difference was determined by a one-way ANOVA, followed by the Tukey's Multiple Comparison test. Statistical significance was defined as a *P-*value ≤0.05.

## Results

In control group, the livers showed normal lobular architecture with central veins and radiating hepatic cords. High dose use of MDMA caused severe hepatic damages such as inflammation and significant hepatocyte damage but in experimental group (200 mg/kg PTX) the number of damaged hepatocytes was significantly decreased compared to the other groups except control (*P*=0.99) (Figure 2). 

TUNEL staining which detects DNA damage characteristic of apoptosis was carried out after acute administration of MDMA and PTX.

**Figure 3:**
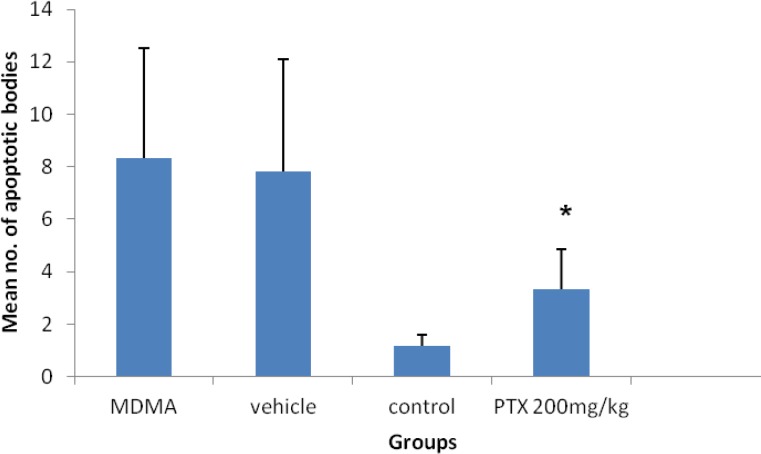
Comparison of apoptotic bodies between groups in TUNEL assay. The apoptotic bodies were significantly decreased by 200 mg/kg pentoxifylline treatment in the liver. *P≤0.05, statistically different from 3,4-Methylenedioxymethamphetamine and vehicle groups

The number of TUNEL-positive cells of liver was significantly decreased in experimental group compared to ecstasy (*P*=0.037) and vehicle groups (*P*=0.042) after acute administration of MDMA. The apoptotic bodies were significantly decreased by PTX treatment in the liver. There was no significant difference between the number of apoptotic bodies in experimental and control (*P*= 0.674) groups (Figure 3).

**Figure 4:**
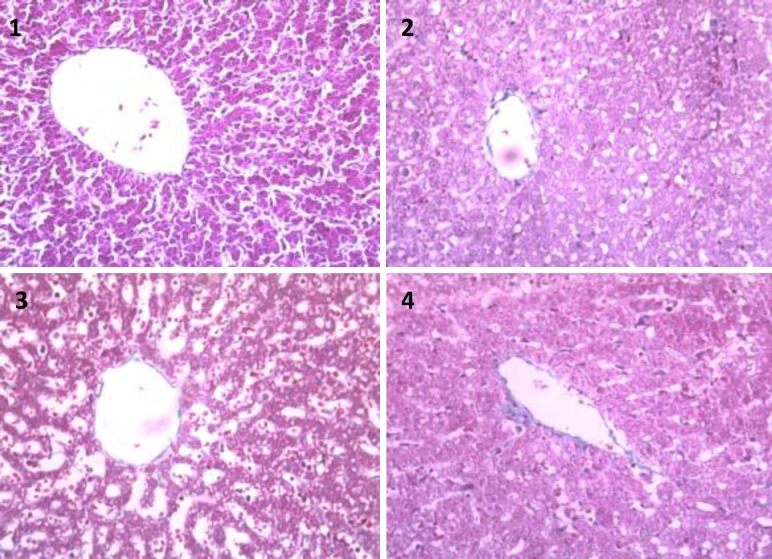
Masson's trichrome stainings of liver sections 1) control group. Fibrosis and collagen fibers could not be seen, 2) Liver tissue slice in experimental group 1. Strands of blue collagen are little seen among hepatocytes, 3) ecstasy group. Blue collagen fibers increasingly are seen among hepatocytes, 4) vehicle group. Blue collagen fibers among hepatocytes are seen. (×400

Hepatic fibrosis was demonstrated by the Masson's trichrome staining (Figure 4). In the present study, the effects of pentoxifylline on the liver fibrosis induced by MDMA have been determined. The rat livers in the experimental group (PTX treated) showed less collagen deposition, fibrosis and damaged hepatocytes compared to vehicle and ecstasy groups (*P*≤0.05). Moderate fibrosis was observed in MDMA group compared to control group, whereas fibrosis and excessive collagen deposition was markedly suppressed in experimental group (Table 1).

## Discussion

MDMA, a methamphetamine derivative known as ‘ecstasy', and other drugs with similar effects are widespread in the world. Ecstasy can be incriminated in causing the liver damage. Many studies showed that the hepatotoxicity induced by MDMA is a consequence of the metabolism of MDMA (26).

Liver fibrosis and its end-stage disease, cirrhosis, are outcomes of many chronic liver diseases, such as viral hepatitis and of alcohol consumption and drug abuse (27).

It has been reported that liver toxicity is one of the consequences of ecstasy abuse. Various factors may play role in ecstasy-induced hepatotoxicity, namely its metabolism, the increased efflux of neurotransmitters, the oxidation of biogenic amines, and hyperthermia (7). MDMA undergoes extensive hepatic metabolism that produce reactive metabolites which form adducts with intracellular nucleophilic sites. Consumption of MDMA involves the production of TNF-α and promote multiple mechanisms to initiate apoptosis in hepatocytes (7)

Many studies have indicated that each of the amphetamine derivatives have direct effects including the induction of apoptosis by phosphorylation (inactivation) of Bcl-2 in MDMA-exposed tissue (28, 29).

The mechanism of hepatic injury induced by MDMA administration is unclear but a spectrum of severity seems to exist as assessed with histological changes varying from a mild to moderate lobular hepatitis to features of massive hepatic parenchymal collapse with areas of nodular regeneration. (30)

Therapeutic effect of pentoxifylline on hepatic lesions induced by ecstasy has not been studied but some studies have shown that pentoxifylline is used for the treatment of liver lesions caused by alcohol (31, 32), liver lesions after surgery (33, 34), bacterial-induced hepatopathy (35, 36) and many other types of liver damage.

 The beneficial effects are believed to occur through various mechanisms such as inhibition of phosphodiesterases, increased cAMP levels and downregulation of TNF-α, IL-1, IL-6, transforming growth factor-beta (TGF-β), interferon-gamma (IFN-γ), stellate cell activation and procollagen-I mRNA expression (23).

Ecstasy-induced apoptosis plays a major role in liver injury and is often seen with inflammation and fibrosis (37). Pentoxifylline can inhibit TNFα by reducing the transcription of its gene and also can reduce inflammation by affecting cytokine/ chemokine pathway (38).

 In this study, we investigated the effect of pentoxifylline on liver lesions in Wistar rats. For the first time in 1991, the therapeutic effect of pentoxifylline on hepatorenal syndrome was studied (39). Later, the beneficial effect of this drug was shown on alcoholic hepatitis by some researchers (40). PTX has been proposed as an effective strategy in treating alcoholic hepatitis, and even in reduction in secondary complications such as hepatic encephalopathy syndrome and hepatorenal syndromes (31, 41). Anti-fibrotic effect of pentoxifylline due to inhibition of profibrogenic cytokine and procollagen I expression is shown by some studies (42). Pentoxifylline can decrease the AST (Aspartate aminotransfrase) and ALT (Alanin aminotransfrase) levels and its anti-TNF-α effect also improves symptoms of liver tissue in patients with NALFD (Non-alcoholic steatohepatitis)/NASH Non-alcoholic fatty liver disease (43).

The results of this study showed decrease in apoptosis and consequently anti-fibrotic properties of pentoxifylline after ecstasy administration. 

## Conclusion

The present study indicates that the number of apoptotic bodies have been decreased effectively after treatment with 200 mg/kg dose of pentoxifylline. It has been concluded that pentoxifylline can reduce ecstasy-induced hepatic lesions and fibroses in Wistar rats.
